# The Temporal Relationship Between Local School Closure and Increased Incidence of Pediatric Diabetic Ketoacidosis

**DOI:** 10.3389/fped.2022.812265

**Published:** 2022-03-11

**Authors:** Casey K. McCluskey, Janine E. Zee-Cheng, Margaret J. Klein, Matthew C. Scanlon, Alexandre T. Rotta, Kenneth E. Remy, Christopher L. Carroll, Steven L. Shein

**Affiliations:** ^1^Department of Pediatrics, West Virginia University School of Medicine, Morgantown, WV, United States; ^2^Department of Pediatrics, Indiana University School of Medicine, Indianapolis, IN, United States; ^3^Department of Anesthesiology and Critical Care, Children's Hospital Los Angeles, Los Angeles, CA, United States; ^4^Department of Pediatrics, Medical College of Wisconsin, Milwaukee, WI, United States; ^5^Department of Pediatrics, Duke University School of Medicine, Durham, NC, United States; ^6^Department of Pediatrics and Internal Medicine, Washington University in St. Louis, St. Louis, MO, United States; ^7^Department of Pediatrics, Connecticut Children's, Hartford, CT, United States; ^8^Department of Pediatrics, Rainbow Babies and Children's Hospital, Cleveland, OH, United States

**Keywords:** diabetic ketoacidosis, pediatrics, critical care, endocrinology, school closure

## Abstract

**Importance:**

The incidence of pediatric diabetic ketoacidosis (DKA) increased early in the COVID-19 pandemic, but the relative contribution of behavioral changes and viral-related pathophysiology are unknown.

**Objective:**

To evaluate the relationship between school closure date and onset of increased DKA to help clarify the etiology of the increased incidence.

**Design:**

A multi-center, quality-controlled Pediatric Intensive Care Unit (PICU) database was used to identify the number of admissions to a participating PICU with DKA on each calendar day from 60 days before local school closure to 90 days after, and compared to baseline data from the same periods in 2018–2019. Interrupted time series and multiple linear regression analyses were used to identify admission rates that differed significantly between 2020 and baseline.

**Setting:**

Eighty-one PICUs in the United States

Participants: Children ages 29 days to 17 years admitted to a PICU with DKA

Exposures: Statewide school closure

Main outcome/measure: Rate of admission to the PICU for DKA.

**Results:**

There were 1936 admissions for children with DKA in 2020 and 1795 admissions/year to those same PICUs in 2018-2019. Demographics and clinical outcomes did not differ before school closure, but pandemic-era patients were less often white and had longer hospital length of stay in the post-school closure period. The difference between 2020 admissions and 2018-2019 admissions was not different than zero before school closure, and significantly higher than zero after school closure, but was significantly increased in 2020 at >30 days after school closure (*p* = 0.039).

**Conclusions/Relevance:**

An increase in pediatric DKA admissions began one month after school closures. Given that behavioral changes started near school closure dates and viral activity peaked weeks after, this suggests that behavioral factors may not be the primary etiology and it is possible that SARS-CoV-2 infection may have direct effects on pediatric DKA.

## Introduction

Higher incidence of diabetic ketoacidosis (DKA) early in the COVID-19 pandemic was reported in the United Kingdom and in the United States (US) (1, 2), but the relative contributions of societal influences (e.g., reduced access to medical care, hesitancy to seek care) and viral infection are unclear. Prior studies have compared the incidence of pediatric DKA in a discrete epoch (e.g., March–April 2020) to preceding years (1, 2). A more longitudinal and granular analysis of daily data may help clarify the etiologies of this rising incidence.

Societal mobility, vaccination rates, sick visits to the pediatrician, and emergency room utilization all decreased concurrently with school closures and other measures aiming to mitigate the pandemic, suggesting that DKA rates should increase near that time if primarily driven by behavioral changes (3–6). Alternatively, a more delayed increase in DKA would be expected if viral factors predominate, since COVID-19 incidence peaked weeks after school closures (7). We evaluated the relationship between school closure date and the number of children admitted with DKA to Pediatric Intensive Care Units (PICUs) across the US.

## Methods

With approval from the Institutional Review Board of Connecticut Children's Medical Center, we queried the Virtual Pediatric Systems (VPS) database (Virtual Pediatric Systems, LLC, Los Angeles, CA). VPS includes quality controlled, patient-level data for all admissions to participating PICUs. VPS neither endorsed nor restricted our interpretation of these data.

Children ages 29 days to 17 years with a primary diagnosis related to DM or DKA were included. Only centers participating in VPS in all of 2018–2020 were included. Demographics, PRISM-3 (Pediatric RISk of Mortality) score, length of stay (LOS), and mortality were collected. To protect privacy, VPS reports age as a categorical variable. To standardize PICU admission date relative to regional COVID spread, the number of days between the admission date of each patient and that state's school closure date [as reported on each state's website (Supplementary Table 1)] were calculated. For example, an admission date of March 2, 2020 was converted to “-11” if the state's school closure date was Friday, March 13, 2020. For admissions in 2018-2019, the reference date (Day 0) was the same calendar date as school closure (e.g., March 13, 2018 or March 13, 2019), though it was shifted by two days for 2019 and three days for 2018 so the day of the week aligned for comparison purposes. Using the example above, a child admitted from the same state would have a reference day (Day 0) of Friday, March 15, 2019 or Friday, March 16, 2018. For all three years, only admissions that occurred between−60 and +90 relative to Day 0 of that year were included for analysis.

Demographics and outcomes were compared with Chi-squared, Fisher's exact and Wilcoxon rank-sum tests. Admission rate over time was analyzed in two ways. First, for each admission day, the number of admissions in 2018–2019 was subtracted from the number of admissions in 2020. Interrupted time series analysis was used to test if this “difference in admissions from baseline” changed significantly at the time of school closure. Second, the raw number of admissions for both 2018-2019 and 2020 were divided into pre-school closure (admission day <0), early post-school closure (0–30; chosen *post-hoc* based on visual inspection of the data) and late post-school closure (>30) epochs. For each epoch, a multiple linear regression model including study year (2020 vs. 2018–2019), days from school closure, and their interaction was constructed. An interaction term with *p* < 0.05 indicated a significant difference in the rate of admissions in 2020 versus baseline. All analyses were performed using SAS v9.4 (SAS Institute Inc., Cary, NC). Data are reported as median (interquartile range) and frequency (percentage).

## Results

There were 1,936 episodes of children admitted with DKA to one of 81 US PICUs in 2020 and 1795/year DKA episodes to those same PICUs in 2018-2019. Demographics and outcomes did not differ between study years during the pre-school closure period (Table 1). In the post-school closure period, pandemic-era patients were less often white and had longer hospital LOS.

**Table 1:** Demographics.

		**2018 and 2019 weighted averages**		**2020^a^**		**Pre vs. pre *P*-value^b^**	**Post vs. post *P-*value^b^**
		**Pre-school closure**	**Post-school closure**	**Pre-school closure**	**Post-school closure**		
		***n* = 738.5**	***n* = 1056.5**	***n* = 700**	***n* = 1236**		
**Age**							
Infant 29 days to <2 years	*n* (%)	19.5 (2.64%)	39 (3.69%)	17 (2.43%)	30 (2.43%)	0.96	0.0017
Child 2 years to <6 years	*n* (%)	61 (8.26%)	83.5 (7.90%)	56 (8.00%)	94 (7.61%)		
Child 6 years to < 12 years	*n* (%)	230 (31.14%)	285.5 (27.02%)	212 (30.29%)	421 (34.06%)		
Adolescent 12 years to < 18 years	*n* (%)	428 (57.96%)	648.5 (61.38%)	415 (59.29%)	691 (55.91%)		
**Female**	*n* (%)	378 (51.18%)	546 (51.68%)	358 (51.14%)	632 (51.13%)	0.99	0.79
**Race** White (vs. non-white)	*n* (%)	328.5 (44.48%)	466 (44.11%)	332 (47.43%)	488 (39.48%)	0.26	0.0251
**ICU length of stay**, days	Median (Q1, Q3)	0.91 (0.67, 1.21)	0.91 (0.70, 1.20)	0.89 (0.68, 1.29)	0.92 (0.69, 1.31)	0.50	0.14
**Hospital length of stay**, days^c^	Median (Q1, Q3)	2.18 (1.55, 3.09)	2.10 (1.51, 3.06)	2.18 (1.57, 3.04)	2.30 (1.66, 3.15)	0.73	0.0052
**PRISM-3 predicted length of stay, days**	Median (Q1, Q3)	1.36 (0.59, 1.80)	1.12 (0.59, 1.61)	1.36 (0.59, 1.80)	1.36 (0.59, 1.80)	0.41	0.0049
**ICU mortality**	*n* (%)	0.5 (0.07%)	2.5 (0.24%)	1 (0.14%)	5 (0.40%)	>0.99	0.73

a *Raw counts*.

b *P-values based on the Chi-squared or Fisher's Exact test (categorical; non-integer numbers were rounded up when the Fisher's Exact test was needed) or the Wilcoxon Singed-rank test (continuous)*.

c *Hospital LOS missing in 15 cases*.

**Figure 1:**
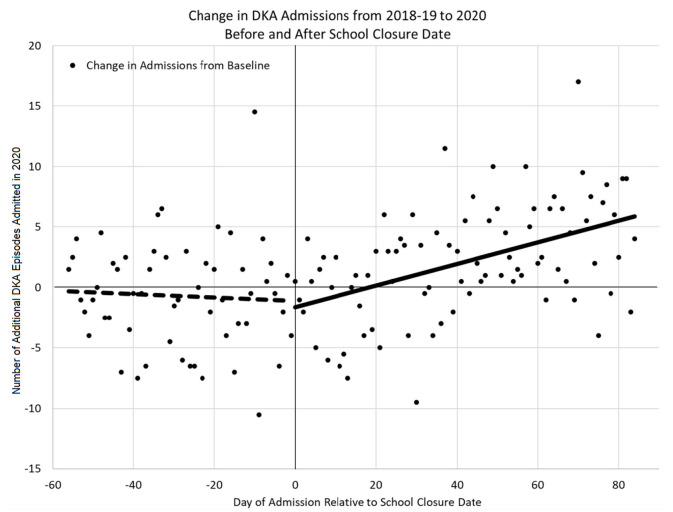
Change in admissions due to diabetic ketoacidosis in 2020 compared to 2018–2019.

**Figure 2:**
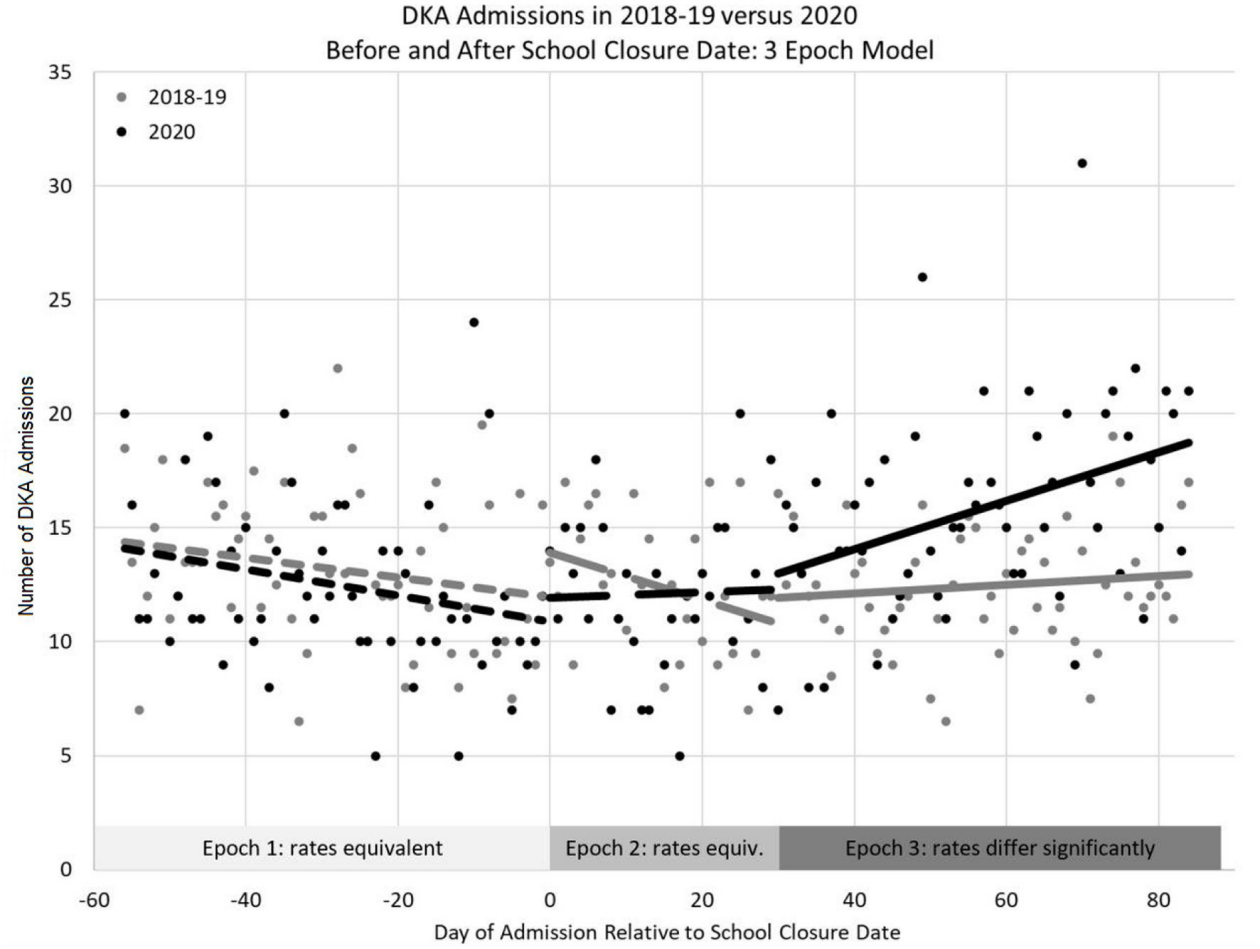
Number of DKA episodes admitted to the Pediatric Intensive Care Unit between 2020 and baseline years (2018–2019 average) relative to school closure date using a 3-epoch model (pre-school closure, early post-school closure, and late post-school closure).

The change in DKA admissions from baseline is shown in Figure 1. The rate of admissions did not differ significantly from zero in the pre-closure period (*p* = 0.684), but did significantly increase in the post-closure period [$\hat{b}$ = 0.09 (95% CI: 0.05–0.13), *p* < 0.001]. Figure 2 shows the actual number of children admitted each study day. The rates of DKA admissions did not differ between study years for either the pre-school closure (interaction *p* = 0.730) or the early school closure (interaction *p* = 0.595) periods. In the late school closure period, the rate of admissions increased significantly faster in 2020 (interaction *p* = 0.039).

## Discussion

We found an increased incidence of pediatric DKA beginning approximately one month after local school closure. One possible explanation is that acute COVID-19 triggered DKA in children with already-established DM as other viral infections commonly do (8). COVID-19 incidence increased for 2–3 weeks following school closure, but we observed no increase in DKA during this time, suggesting that acute viral infection was not the key driver (7). Alternatively, reduced access to school-based care, stress, and changes in diets occurred during the pandemic's early stages, which can worsen glycemic control and increase the risk of DKA (8). However, Italian children actually had improved glycemic control during their lockdowns (9), and we would expect these etiologies to have increased DKA incidence sooner after school closures if they were the leading factors. It is also possible that behavioral variables may have been more favorable for DM control in the first weeks after school closure (e.g., parents working from home initially then returning to the office), underscoring that an epidemiologic study like ours cannot absolutely prove nor refute the underlying mechanisms.

Viral infections may also precipitate development of DM in previously healthy children (10). SARS-CoV-2 enters cells via the angiotensin-converting enzyme 2 (ACE2) receptor, which is abundant in the pancreas, leading to speculation that it may contribute to DM pathogenesis (11). An increase in the number of previously healthy children presenting with new-onset DM during the first three months of lockdown was observed in one region of London, but national studies from Germany and Italy did not find similar increases over the first two months of their lockdowns (12–14). Our data suggest that a longer evaluation period may be necessary to identify changes in DM epidemiology. Further work is needed to establish if increases in new-onset DM as a postviral infection related phenomenon drove the increase in DKA admissions especially given recently emerging data from the first 12 months of the pandemic that showed a statistically significant increase in the number of children with new-onset DM in the greater San Diego area (15).

Delays in seeking medical care could increase the rate at which hyperglycemic episodes progress to DKA. This may explain the increase in severe DKA at initial presentation of DM observed in Europe (13, 14). However, the time course at which we observed an increase in DKA does not align with other reports of behavioral changes during this time. In-restaurant dining and workplace presence began to decrease 1–2 weeks before school closure (4). Childhood immunization rates in New York City, sick visits in Scotland and emergency room visits all decreased the same week as local school closures (3, 5). Though motives of preventative and emergency care may differ, this suggests that parental apprehension likely peaked closer to school closure than the time at which we observed the increase in DKA, and that it is unlikely to be the key driver of our findings.

While this is the most comprehensive evaluation of pediatric DKA admissions during the early COVID-19 pandemic to date, several limitations warrant consideration. First, insufficient laboratory data are available in VPS to confirm if all subjects had DKA, though >97% of subjects had “DKA” in their primary diagnosis. Second, VPS only collects data from PICUs, so hospitals that also treat cases of DKA on the general ward may not have all patients captured, and our findings may not be generalizable to non-severe DKA or patients cared for outside the PICU. However, since all centers contributed both baseline and pandemic data, this should have little impact on our comparative analyses, though it is possible that ICU admission criteria may have varied at different stages of the pandemic. Third, insufficient data were available to report the rate of new-onset DM in this cohort and we unfortunately could not reliably distinguish new-onset DM from exacerbation of a known condition. We also did not have reliable COVID status available to us in this dataset. Fourth, our dataset included all children with a primary diagnosis related to DM or DKA, which did include both Type 1 DM and Type 2 DM. While the majority of patient's had Type 1 DM, patients with Type 2 DM may take longer to develop metabolic derangements. Fifth, we assigned a single school closure date to all children in the same state, but individual school districts may have closed prior to the statewide closure.

In conclusion, the increase in children admitted to a PICU with DKA started approximately one month after local school closure. This lag suggests that behavioral changes may not be the primary etiology and that it is possible SARS-CoV-2 may have direct effects that increase DM and DKA among children, as suggested by others, (15) though further studies are needed to better adjust for confounding factors associated with the lockdowns.

## Data Availability Statement

The raw data supporting the conclusions of this article will be made available by the authors, without undue reservation.

## Ethics Statement

The studies involving human participants were reviewed and approved by Connecticut Children's Medical Center. Written informed consent from the participants' legal guardian/next of kin was not required to participate in this study in accordance with the national legislation and the institutional requirements.

## Author Contributions

CM, JZ-C, CC, and SS carried out the lit review and wrote the manuscript. MK, SS, and MS performed the statistical analysis. All authors contributed to the manuscript revisions, conceived and designed the study, and read and approved the final version of the manuscript.

## Conflict of Interest

The authors declare that the research was conducted in the absence of any commercial or financial relationships that could be construed as a potential conflict of interest.

## Publisher's Note

All claims expressed in this article are solely those of the authors and do not necessarily represent those of their affiliated organizations, or those of the publisher, the editors and the reviewers. Any product that may be evaluated in this article, or claim that may be made by its manufacturer, is not guaranteed or endorsed by the publisher.
