# Supplementary material for: The Temporal Relationship Between Local School Closure and Increased Incidence of Pediatric Diabetic Ketoacidosis
Source: Front Pediatr. 2022 Mar 11;10:812265. doi: 10.3389/fped.2022.812265 (PMC8963207; doi:10.3389/fped.2022.812265)
Supplement: Supplementary file 1 [file Data_Sheet_1.PDF]

Supplemental Table 1

State	School closure date
Michigan	3/12/2020
Louisiana	3/13/2020
West Virginia	3/13/2020
North Carolina	3/14/2020
Arizona	3/15/2020
Hawaii	3/15/2020
South Carolina	3/15/2020
Alaska	3/16/2020
Connecticut	3/16/2020
Delaware	3/16/2020
Florida	3/16/2020
Illinois	3/16/2020
Iowa	3/16/2020
Kentucky	3/16/2020
Maryland	3/16/2020
Mississippi	3/16/2020
Montana	3/16/2020
Nevada	3/16/2020
New Hampshire	3/16/2020
New Mexico	3/16/2020
North Dakota	3/16/2020
Ohio	3/16/2020
Oklahoma	3/16/2020
Oregon	3/16/2020
Pennsylvania	3/16/2020
Rhode Island	3/16/2020
Utah	3/16/2020

State	School closure date
Pennsylvania	3/16/2020
Rhode Island	3/16/2020
Utah	3/16/2020
Virginia	3/16/2020
Arkansas	3/17/2020
Massachusetts	3/17/2020
South Dakota	3/17/2020
Washington	3/17/2020
Alabama	3/18/2020
Georgia	3/18/2020
Minnesota	3/18/2020
New Jersey	3/18/2020
New York	3/18/2020
Vermont	3/18/2020
Wisconsin	3/18/2020
Missouri	3/19/2020
California	3/20/2020
Indiana	3/20/2020
Tennessee	3/20/2020
Texas	3/20/2020
Colorado	3/23/2020
Kansas	3/23/2020
Wyoming	3/23/2020
DC	3/24/2020
Idaho	3/24/2020
Nebraska	4/1/2020
Maine	4/2/2020
